# The differential mediating roles of resilience in the relationship between meaningful living and stress among college students during the COVID-19 pandemic

**DOI:** 10.1038/s41598-023-38975-0

**Published:** 2023-07-19

**Authors:** Tamadhir Al-Mahrouqi, Moon Fai Chan, Maryam Al-Mukhaini, Manar Al Shehi, Salim Al-Huseini, Firdous Jahan, Naser Al Balushi, Sathiya M Panchatcharam, Mohammed Al Alawi

**Affiliations:** 1 Psychiatry Residency Training Program, Oman Medical Specialty Board, Muscat, Oman; 2 Department of Family Medicine and Public Health, Sultan Qaboos University, Muscat, Oman; 3 College of Pharmacy, National University of Science and Technology, Muscat, Oman; 4 College of Medicine and Health Sciences, National University of Science and Technology, Muscat, Oman; 5 Behavioral Medicine Department, Sultan Qaboos University Hospital, Sultan Qaboos University, Al Khoud, Muscat, Oman; 6 Research Department, Oman Medical Specialty Board, Muscat, Oman

**Keywords:** Psychology, Medical research

## Abstract

The current literature, mostly Euro-American based, indicates that the presence of meaning in life (MIL) improves resilience and lowers stress. However, the differential mediating roles of resilience in the relationship between the search for and presence of MIL, and stress have not been explored. This study aimed to investigate the differential mediating roles of resilience in the relationship between the presence of and search for MIL, and stress among Omani college students amid the COVID-19 pandemic. This cross-sectional study consisted of the Brief Resilience Scale, Perceived Stress Scale 4, and Meaning in Life Questionnaire, as well as socio-demographic questions. A path analysis model was used to examine the hypothesis. A total of 970 Omani college students responded to the questionnaire. Findings indicate that searching for MIL was significantly associated with a high level of stress directly (β = 0.023; *p* < 0.001) and indirectly, through a negative effect on resilience (β =  0.006; *p* < 0.001). Conversely, the presence of MIL was significantly associated with a decreased level of stress directly (β = − 0.045; *p* < 0.001) and indirectly via a positive effect on resilience (β = − 0.151; *p* < 0.001). In keeping with the proposed hypothesis, this study contributes to the current knowledge, by extrapolating the effect of searching for MIL on resilience and stress, and culturally re-contextualizing MIL research. University counseling centers could adopt meaning-based strategies to mitigate stress by promoting meaningful living and resilience.

## Introduction

The COVID-19 pandemic has significantly impacted the education system and learning approaches of 1.6 billion students across 200 countries, many of whom experienced added stress from being quarantined^1^. This stress can result from various pandemic-related factors, such as fear of infection, social isolation, disrupted sleep and eating patterns, financial strain, difficulty concentrating, and increased academic workload^2,3^. When we consider all the challenges and situations that have arisen due to the COVID-19 pandemic^2^, it is likely that university students in Oman may have experienced significant distress. In light of this, it is worth noting that the pandemic has been viewed by many as an existential threat^4^, which makes having a sense of meaning in life (MIL) and resilience crucial for college students to cope with the ongoing stress.

Within the above context, the present paper proposes a mediational model linking the three constructs: MIL, resilience, and stress. The model draws on the three constructs' existing theoretical and conceptual frameworks, which will be detailed next.

Stress is defined as the sensation of emotional or physical tension and can lead to academic, social, and occupational dysfunction^5^. Although stress can result from positive and negative aspects of an individual's life, high levels of stress are generally associated with negative psychological and physical complications^6^. Studies suggest that stress can worsen many diseases and pathological conditions, making it a triggering or aggravating factor^7^.

The present study defines stress based on Lazarus and Folkman’s transactional model^8^, which highlights the importance of the person-environment transaction and cognitive appraisal processes. The individual assesses the relevance of stressors (primary appraisal) and their resources to cope (secondary appraisal) before reacting. Cohen developed the Perceived Stress Scale (PSS) based on this model, which is widely used to measure the degree of stress individuals perceive in general or during specific experiences, such as the current pandemic's effect on college students^6^. According to this model, the impact of stress on individuals varies based on their level of resilience^5^.

Resilience plays a crucial role in determining how stress affects individuals. It refers to the ability to bounce back from adversity and thrive in challenging situations like the COVID-19 pandemic^9^. Studies have suggested that resilience acts as a psychological protective factor for students, with higher levels inversely correlated to acute^10,11^. The individual’s overall level of resilience is affected by biological, psychological, economic, familial, cultural, societal and existential factors^10–13^. Family and cultural background can provide a supportive environment and influence an individual’s coping strategies^13^. Societal and economic factors, such as access to resources and financial stability, can also impact resilience^14^. Spirituality and religious beliefs can serve as a source of strength and comfort during difficult times^15^. Psychological factors, such as having a positive outlook and strong self-esteem, are also important for resilience^16^. Finally, a sense of purpose and meaning in life, referred to as existential factors, can contribute to resilience^17^. In this study, meaning in life is being examined as an existential force that could potentially affect the resilience of college students during COVID-19 tribulations^4^.

A sense of MIL comprises the individual’s ability to comprehend and understand their sense of self, life, and their ability to define purpose and significance in life^18^. MIL is particularly important during the COVID-19 pandemic, as it predicts resilience and psychological health in young adults^19^.

The present study builds on the conceptual model of meaning in life proposed by Steger et al.^20^ who formulated The Meaning in Life Questionnaire (MLQ). MLQ assesses two different dimensions of the construct: the presence of MIL and the search for MIL^3^. The presence of MIL refers to how individuals perceive their lives as being significant and meaningful, while the search for MIL refers to how much individuals are actively engaged in searching for meaning in their lives^20^. Several studies conducted in the United States among undergraduate students reported that having MIL was associated with lower levels of perceived stress and better mental health outcomes^21,22^.

Although the relationship between MIL, resilience, and perceived stress has been thoroughly studied, there is a lack of research on the mediating role of resilience between a search for MIL and perceived stress. Additionally, there is limited literature on this topic in the Arabic and Islamic world. This study aims to investigate the mediating roles of resilience in the relationship between the presence and search for MIL and perceived stress among Omani university students. The primary hypothesis suggests that the presence of MIL would be associated with lower levels of stress directly and indirectly through the positive effect on resilience and that searching for MIL would be associated with an elevated level of stress directly and indirectly by lowering the levels of resilience.

That mentioned, next, we describe the methods used to collect and analyze data. In the “Results” section, we present our findings in light of the proposed hypothesis. In the “Discussion” section, we analyze the results within the broader theoretical frameworks and existing literature. Finally, we conclude the paper with a summary of the main findings and the implications from theoretical, and managerial standpoints, as well as suggestions for future research.

## Methods

### Study design and setting

This was a cross-sectional study that included students at the National University of Science and Technology in Oman, a multi-campus facility with a total of 2766 students enrolled in the academic year 2021–2022. All four campuses were invited to participate in the study: the College of Engineering, the College of Medicine, the College of Pharmacy, and the School of Foundation Studies. Data collection was from 7 to 29 April 2021.

### Participant selection and data collection

This study utilized a self-administered online questionnaire that was distributed to all students enrolled in the National University of Science and Technology for the academic year 2021–2022 via institutional emails. Students who refused to sign the electronic informed consent form or filled incomplete questionnaire were excluded from the study. The study was approved by the Research and Ethics Committee at the National University of Science and Technology, and the study procedures complied with ethical standards. The research method was performed in accordance with relevant guidelines/regulations and the Declaration of Helsinki recommendations.

Data were collected through a combination of convenience sampling and snowballing methods, resulting in a response rate of 35%. The surveys were distributed through institutional emails and batch and class representatives were asked to explain the study purpose to their fellow colleagues. The university counseling center was also engaged to encourage students to participate. Follow-up reminders were sent to all participants through email to increase the response rate.

### Measurement outcomes

#### The sociodemographic questionnaire

The demographic information of each participant, including, age, gender, academic major, academic year, and presence of a physical or mental illness was obtained. Academic major was categorized into three main majors: pharmaceutical sciences, doctor of medicine, and engineering. Also, the questionnaire collected information about whether they are currently living with family, and friends or living alone, and the presence of any financial strains. A participant was considered to have a chronic physical illness if they checked off at least one chronic illness from a long list of diseases on the questionnaire. The survey covers gastrointestinal (such as celiac disease), neurological (such as migraine), endocrine (such as diabetes), hematological (such as sickle cell disease), respiratory (such as asthma), and dermatological illnesses (for example, eczema). A participant was considered to have a mental illness if they reported having at least one condition from a comprehensive list of mental illnesses in the online survey. Anxiety disorders, depressive disorders, eating disorders, personality disorders, and bipolar disorders are all covered in the survey.

#### Measurement of resilience

The Brief Resilience Scale (BRS) was used, a self-reported questionnaire consisting of 6 questions assessing the unitary construct of resilience^23^. Participants are asked to rate their level of agreement or disagreement with each question on a 5-point Likert scale, ranging from 1 = strongly disagree to 5 = strongly agree. Questions 1, 3, and 5 are positively phrased while questions 4 and 6 are negatively phrased and are reversed coded. The total score is obtained as the mean of all 6 questions. Scores of 1.00–2.99 correspond to low resilience, 3.00–4.30 to normal resilience, and 4.31–5.00 to high resilience^23^. A systematic review reported the internal consistency and Cronbach’s alpha of 0.69^24^ and our study reported a Cronbach’s alpha of 0.5, likely due to the small number of scale items and heterogeneity of latent factors. No permission was required to use the scale for research purposes.

#### Measurement of stress

The Perceived Stress Scale 4 (PSS-4) was used as a reduced version of the 14-item Perceived Stress Scale^25^. This is a self-reported questionnaire focused on general questions rather than specific experiences, consisting of 4 questions, two assess the participants perceived stress while the other two measure coping strategies towards stress. Participants are asked to rate their perceived stress level within the last month based on their thoughts and feelings on a 5-point Likert scale, ranging from 0 = never to 4 = very often. The total score is obtained as the sum of all 4 questions, with scores ranging from 0 to 16. The Higher total scores are correlated with higher perceived stress^25^. The PSS-4 scale offers an obvious benefit in terms of time to complete and convenience of use, and ease of completion via the Internet^26,27^. A systematic review of 6 studies reported that the internal consistency reliability measured by Cronbach’s alpha was 0.60^25^, and in the current, this was 0.42, not unexpected when using the 4-item version of the PSS. No permission was required to use this scale for research purposes.

#### Measurement of the meaning in life

The Meaning in Life Questionnaire (MLQ) is a self-reported questionnaire consisting of 10 questions^20^. Questions 1, 4, 5, 6, and 9 assess the presence of meaning in the participants’ lives, while questions 2, 3, 7, 8, and 10 assess how the participants are engaged in searching and finding meaning in their lives. Participants are asked to rate their degree of agreement on a 7-point Likert scale, ranging from 1 = Absolutely untrue to 7 = Absolutely true. Question 9 is reversely coded. The total score is the sum of all questions in each category ranging from 5 to 35, higher scores correlate with higher levels of presence of meaning and searching for meaning in their lives^20^. The MLQ improves on current MIL measures in various ways: there is no item overlap with distress measures, a stable factor structure, better discriminant validity, a simpler format, and the capacity to assess the search for meaning^20^. In this study, the internal consistency was 0.84 and 0.88 on the presence and search scale, respectively, similar to a previous study^28^.

### Sample size calculation

The minimum required sample size was estimated with the formula from Cohen et al*.* (2003, p. 92) for computing power for regression coefficients^29^.

- For a one-predictor ordinary least squares regression with an alpha level of 0.05 and power of 80%, L is equal to 7.85. Based on these values, the minimum sample size needed for 80% to detect the α and β̂ paths when both empirically equal 0.14 is 403.

### Statistical analyses

**Figure 1:**
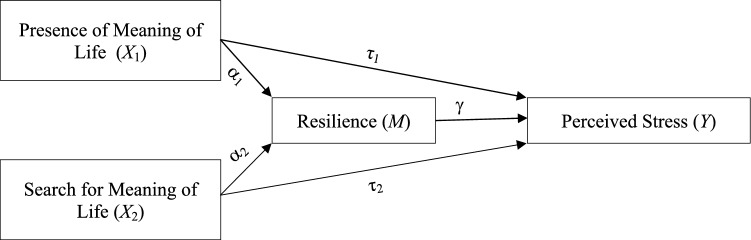
A hypothesized conceptual model for the mediational roles of resilience in the relationship between “presence of” and “search for” MIL and levels of perceived stress.

Descriptive statistics, including frequency, percentage, mean, standard deviation (SD), median, and range, were used to explore the profile of the students according to their demographic and psychological measures. In our conceptual model (Fig. 1), the observed effect of the presence of MIL (X1) and search of MIL (X2) on perceived stress (Y) are called the total effect. The total effect comprised a direct effect pathway of X1 and X2 on Y and a total indirect pathway of X1 and X2 on Y through resilience (M). The PSS-4 measured the perceived stress, resilience was measured by the BRS, and the MLQ measured the presence of MIL and search of MIL. Direct and indirect effects were then explored by mediation analysis: the standardized coefficients β were estimated for each variable with standard errors (SE), z-statistics, and its P values were reported. The model fit was assessed by R2 (coefficients of determination). The full information maximum likelihood approach was used for model estimation. A bootstrap method was used to test the significance of the total and indirect effects across all variables with 5000 bootstrap samples. The coefficients were considered statistically significant if the zero value was out of its 95% confidence intervals. All analyses were performed using the JASP Statistical Package^30^, and a value of p < 0.05 was considered to be statistically significant.

### Consent to publish

The publisher has all authors’ permission to publish research findings in an online open-access publication.

## Results

### Characteristics of the students

A total of 970 students participated in this study. Table 1 shows the demographic and psychological characteristics of the participants. The majority of the participants were female (n = 812) and living with either family or friends (n = 937, 96.6%). Their ages ranged from 15 to 47 years, with an average age of 21.3 ± 3.1 years. Most (n = 580) reported they had close contact with people who tested positive for COVID-19. Some reported that they had financial difficulties (n = 276) and chronic physical illness (n = 125). The average year's program study was 3.1 ± 1.6 years, ranging from 1 to 7 years. Most of the students were studying engineering (45.4%) and medicine (41.0%). Among other psychological measures, 10% reported to be suffering from a mental illness (n = 102). The average resilience score measured by the BRS was 3.0 ± 0.5, ranging from 1.0 to 4.8. The average perceived stress level measured by the PSS-4 was 7.9 ± 2.4, ranging from 0.0 to 15.0. The average scores for the presence of MIL and search of MIL were 24.3 ± 6.9 and 24.9 ± 7.7, respectively.

**Table 1:** Characteristics of the participants (n = 970).

Demographic	n (%)
Gender	
Female	812 (83.7)
Male	158 (16.3)
Age (Years)	
Mean ± SD	21.3 ± 3.1
Median [range]	21.0 [15.0–47.0]
Study program	
Pharmacy	132 (13.6)
Engineering	440 (45.4)
Medicine	398 (41.0)
Year of study	
1	203 (20.9)
2	199 (20.5)
3	181 (18.7)
4	197 (20.3)
5	114 (11.8)
6	49 (6.1)
7	27 (2.8)
Mean ± SD	3.1 ± 1.6
Median [range]	3.0 [1.0–7.0]
Living alone	
Yes	33 (3.4)
No	937 (96.6)
With family	920 (98.2)
With friends	17 (1.8)
Financial difficulties	
Yes	276 (28.5)
No	694 (71.5)
Close contact with people + ve Covid19^a^	
Yes	580 (60.2)
No	384 (39.8)
Chronic physical illness	
Yes	125 (12.9)
No	845 (87.1)
Psychological outcomes	
Mental illness	
Yes	102 (10.5)
No	868 (89.5)
Resilience (BRS)	
Mean ± SD	3.0 ± 0.5
Median [range]	3.0 [1.0–4.8]
Perceived stress (PSS-4)	
Mean ± SD	7.9 ± 2.4
Median [range]	8.0 [0.0–15.0]
Meaning in life (MLQ)	
Presence	
Mean ± SD	24.3 ± 6.9
Median [range]	25.0 [5.0–35.0]
Search	
Mean ± SD	24.9 ± 7.7
Median [range]	27.0 [5.0–35.0]

^a^6 missing data; Brief Resilience Scale (BRS): 6 items, score 1–5/item, ranging from 1.00 to 5.00, higher scores meaning high resilience; Perceived Stress Scale-4 (PSS-4): 4 items, score 0–4/item, ranging from 0 to 16, higher score meaning perceived higher stress; Meaning of Life Questionnaire (MLQ): 10 items, score 1–7/item, two sub-scale: Presence and Search, each scale range 5–35.

### Mediation analysis

**Figure 2:**
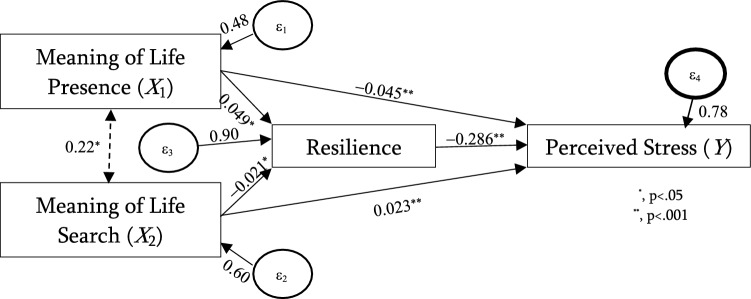
The final model.

Figure 2 illustrates the standardized path estimates and R^2^ of the conceptual model. All the path coefficients were highly significant (*P* < 0.001), indicating that all hypothesized relationships between constructs were supported. Table 2 summarizes the results of the mediation analysis. The direct path from the presence of MIL (β = − 0.045; *p* < 0.001) negatively affected stress. In contrast, the search for MIL (β = 0.023; *p* < 0.001) positively affected stress. Additionally, the presence of MIL has an indirect, statistically significant, negative effect on stress via resilience (β = − 0.151; *p* < 0.001). In contrast, the search for MIL has an indirect, statistically significant, positive effect on stress via resilience (β = 0.006; *p* < 0.001). The total effect of the presence of MIL and search for MIL on stress were − 0.059 (*p* < 0.001) and 0.029 (*p* < 0.001), respectively.

**Table 2:** Direct, indirect, and total effects of search for and presence of MIL and resilience on perceived stress according to the conceptual model.

Model	β (Std.)	SE	z	95% confidence interval (CI)				p-value
				Normal theory (n = 970)		Bias-corrected percentile^a^		
				Lower	Upper	Lower	Upper	
Direct effects								
Presence of MIL (X_1_) → Stress (Y)	− 0.045	0.005	− 9.548	− 0.054	− 0.036	− 0.055	− 0.035	< 0.001
Search for MIL (X_2_) → Stress (Y)	0.023	0.004	5.518	0.015	0.031	0.014	0.031	< 0.001
Indirect effects								
Presence of MIL (X_1_) → Resilience (M) → Stress (Y)	− 0.014	0.002	− 6.980	− 0.018	− 0.010	− 0.019	− 0.010	< 0.001
Search for MIL (X_2_) → Resilience (M) → Stress (Y)	0.006	0.001	4.541	0.004	0.009	0.004	0.010	< 0.001
Total effects								
Presence of MIL (X_1_) → Stress (Y)	− 0.059	0.005	− 12.561	− 0.0068	− 0.0050	− 0.069	− 0.049	< 0.001
Search for MIL (X_2_) → Stress (Y)	0.029	0.004	6.867	0.021	0.037	0.020	0.038	< 0.001

Std., standardized; ^a^, 5000 bootstrap samples; R^2^ = 0.22; MIL, meaning in life.

## Discussion

This study investigated the mediating roles of resilience in the relationship between the presence of and search for Meaning in Life (MIL) and stress among undergraduate Omani students during the COVID-19 pandemic. The proposed hypothesis stated that the presence of MIL would be associated with a high level of stress directly and indirectly through the positive effect on resilience and that searching for MIL would be associated with an elevated level of stress directly and indirectly by lowering the levels of resilience. The results supported the proposed hypothesis and shed light on a novel area of research that has been largely unexplored in Arabic and Muslim cultures.

The results of the first part of the mediational model indicate that the presence of MIL has a significant negative effect on perceived stress, which supports the notion that a meaningful life can contribute to better mental health outcomes. These findings are consistent with previous research indicating that people who have a sense of meaning in their lives have more self-control and report less mental distress^31^. Meaningfulness could have acted directly as a buffer during COVID-19 stress with the presence of meaning and self-control accounting for reduced general mental distress during the pandemic.

The present study also revealed that the presence of MIL predicted resilience and that resilience mediated the effect of meaningful living on perceived stress. Previous research has shown a positive association between the presence of MIL and resilience^19,32^. Therefore, these results underscore the importance of living a life with purpose and meaning in building and promoting resilience among university students. Individuals who experience higher levels of meaningful living are more resilient, can produce various coping strategies when experiencing adversities and hardships, and can manage stressors, which is consistent with previous research^33^.

The second part of the mediational model showed that searching for MIL was associated with higher levels of stress, both directly and indirectly through a negative effect on resilience. To the best of our knowledge, this is the first study to reveal that searching for MIL is associated with lower levels of resilience, which partially explains why searching for MIL is linked to higher levels of perceived stress. This finding expands on Steger’s MIL theory and highlights the complex and dynamic relationship between searching for and the presence of MIL. The pursuit of meaning and purpose in life can have a dual impact on an individual's resilience, and it is crucial to strike a balance in the pursuit of meaning and purpose to cultivate resilience and enhance well-being^20^.

However, prospective studies argued that the search for MIL is the basis for meaningful living. This highlights the complex and dynamic relationship between searching for and the presence of MIL. Indeed, no one is born with a sense of meaning, and those who have meaning in their life have gone through a long and difficult process of learning and discovering^17^. Eventually, once the journey of searching for meaning is fulfilled, it becomes satisfying. Individuals who report having high levels of both presence of and searching for MIL are individuals who already have substantial MIL and continue to pursue more meaning. The search for MIL amongst these individuals was attributed to happy experiences, instead of distressing ones^34^.

Therefore, the pursuit of meaning and purpose in life can have a dual impact on an individual's resilience. While it has the potential to foster a sense of control, increase self-esteem, and promote a positive outlook, it can also lead to decreased resilience in certain circumstances^34^. These include over-focus, resulting in frustration and decreased confidence, rigid expectations that make one more vulnerable to stress and adversity, increased pressure to live up to expectations, and negative rumination that can lead to decreased confidence. Thus, it is crucial to strike a balance in the pursuit of meaning and purpose and be mindful of the potential negative effects to cultivate resilience and enhance well-being^34^.

All in all, this study adds to the growing body of research on the role of MIL in promoting resilience and reducing stress during the COVID-19 pandemic, particularly in Arabic and Muslim cultures. The findings highlight the importance of living a life with purpose and meaning in building resilience and managing stress, while also emphasizing the need to strike a balance in the pursuit of meaning and purpose to cultivate resilience and enhance well-being. These results have implications for interventions aimed at promoting resilience and managing stress during challenging times, such as the COVID-19 pandemic.

### Limitations and future directions

This study has several limitations. First, causal inference is hampered by this study's cross-sectional design. Cohort and experimental study designs would be robust alternatives to decipher causality using mediation analyses. Second, all psychological constructs were measured using self-reported measures, which are subject to self-reporting and social desirability biases. Thirdly, convenience sampling was used to recruit participants. Despite this, the high effective response rate and the large sample size seemed to increase the generalizability of the results. Finally, according to the path coefficients, the effect sizes were small, which is not uncommon when exploring complex constructs. Besides, the measurement errors of the variables in the mediation model should be acknowledged.

To further support our findings, and explore the causal relationship randomized controlled trials (RCT) or longitudinal studies are recommended. Moreover, to investigate the interconnections between psychological resilience, perceived stress, and the pursuit and presence of meaning in life, qualitative or mixed-model methodologies should be considered.

## Conclusions

This study hypothesized a conceptual framework to understand the differential mediating roles of resilience in the relationship between the presence of and searching for MIL and perceived stress among a sample of undergraduate college students in Oman. In agreement with our hypothesis, resilience played a mediating role in the relationship between the presence of and searching for MIL and perceived stress. The results showed that meaningful living has a strong negative predictive effect on perceived stress, as well as a strong positive predictive effect on resilience. In addition, resilience also mediated the effect of meaningful living on perceived stress amongst the study sample. From a theoretical perspective, the present work contributes to the knowledge, by, specifically, extrapolating the effect of searching for MIL on resilience and culturally re-contextualizing MIL research. From managerial and policy-making perspectives, the study results could encourage mental health professionals to consider meaning-based interventions to cultivate resilience and help individuals overcome inevitable stress. Additionally, future studies, based on longitudinal design, are needed to scrutinize the effect of search for meaning on resilience given the scarcity of literature on this topic.

## Data Availability

The research data are available based on a request sent to alalawim@squ.edu.om.
